# Supplementary material for: Regulation of ADP-ribosyltransferase activity by ART domain dimerization in PARP15
Source: Nat Commun. 2025 Oct 29;16:9567. doi: 10.1038/s41467-025-65315-9 (PMC12572374; doi:10.1038/s41467-025-65315-9)
Supplement: Supplementary file 1 — Supplementary Information [file 41467_2025_65315_MOESM1_ESM.pdf]

Supplementary Information accompanying:

Regulation of ADP-ribosyltransferase activity by ART domain dimerization in PARP15

Carmen Ebenwaldner[1], Antonio Ginés García Saura[1], Simon Ekström[2], Katja Bernfur[1], Martin Moche[3], Derek T. Logan[1], Michael S. Cohen[4] & Herwig Schüler[1,*]

[1] Division of Biochemistry and Structural Biology, Department of Chemistry, Lund University, SE-22362, Lund, Sweden

[2] BioMS - Swedish National Infrastructure for Biological Mass Spectrometry, SE-22184, Lund, Sweden.

[3] Protein Science Facility, Department of Medical Biochemistry and Biophysics, Karolinska Institutet, 171 77, Stockholm, Sweden

[4] Department of Chemical Physiology and Biochemistry, Oregon Health and Science University, 3181 SW Sam Jackson Park Rd., L334, Portland, Oregon 97239, United States.

*Correspondence: herwig.schuler@biochemistry.lu.se

Contents

Supplementary Data 2

Supplementary Table 1 2

Supplementary Table 2 3

Supplementary Figure 1 4

Supplementary Table 3 5

Legend – see following page. 6

Supplementary Figure 2 7

Supplementary Table 4 8

Supplementary Figure 3 9

Supplementary Figure 4 10

Legend – see following page. 11

Supplementary Figure 5 12

Supplementary Figure 6 13

Supplementary Table 5 14

Supplementary Figure 7 15

Supplementary Table 6 16

Supplementary Figure 8 17

Supplementary Figure 9 18

Supplementary Figure 10 19

Supplementary References 19

Supplementary Data

Supplementary Table 1. Details on the SEC-RALS/LALS analysis of indicated PARP constructs, related to Figure 1. Source data are provided as a Source Data file.

	PARP15 ART	PARP14 ART	PARP10 ART		PARP15 m2	PARP15 m2-ART
			Peak 1	Peak 2		
***RV (ml)**	16.02	17.34	15.79	16.61	16.66	14.47
**Mw (g/mol)**	47 678	25 903	54 347	31 636	23 071	71 361
**Mp (g/mol)**	47 253	25 695	52 942	30 210	22 365	71 604
**Calculated Mw (g/mol)**	25 402	24 966	24 034	24 034	22 382	47 316
**Mw/Mn**	1.001	1.001	1.003	1.005	1.001	1.003
**IVw (dL/g)**	0.0314	0.0219	-	0.0214	0.0419	0.0462
**Rhw (nm)**	2.89	-	-	2.17	2.48	3.73
**Frac. Of sample (%)**	100	100	9	91	100	100
**Measured conc. (mg/ml)**	0.65	0.37	0.49	0.49	0.74	0.55
**Recovery (%)**	87.0	106	74.7	74.7	98.4	73.3

*RV: retention volume measured at peak maximum
Mw: weight average molecular weight from the molecular weight distribution
Mp: molecular weight at the peak apex
Calculated Mw: calculated using http://web.expasy.org/protparam/ based on the amino acid sequence of the expression constructs
Mw/Mn: dispersity
IVw: weight average intrinsic viscosity
Rhw: weight average hydrodynamic radius
Frac. of sample: fraction of the sample in this peak expressed as a percent of the total area of all the analyzed peaks
Measured conc.: measured concentration of the peak
Recovery: recovery of sample based on RI peak area and input dn/dc compared to the input sample concentration
Definitions from the OMNISEC System User Guide (Malvern Panalytical, MAN0550-06-EN)

Supplementary Table 2. Discrete Model Genetic Algorithm fitting results of PARP15 ART sedimentation velocity – analytical ultracentrifugation (SV-AUC) data. The SV-AUC experiment was performed at 0.5 μM PARP15 ART at 58,000 rpm and measured at 220 nm. The RMSD of the fit is 0.0021678. 95% confidence intervals were determined by Monte Carlo analysis with 100 iterations.

**Parameter**	**Mean Value**	**95% Confidence (low)**	**95% Confidence (high)**
Monomer sedimentation coefficient	2.1385e-13	2.0389e-13	2.2380e-13
Monomer diffusion coefficient	8.2310e-07	6.9467e-07	9.5153e-07
Monomer MW (Da)	25422	(Fixed)	
Monomer frictional ratio (f/f_0)	1.3357	1.1099	1.5615
Dimer sedimentation coefficient	4.0016e-13	3.7738e-13	4.2294e-13
Dimer diffusion coefficient	7.6819e-07	7.0773e-07	8.2865e-07
Dimer MW (Da)	50844	(Fixed)	
Dimer frictional ratio (f/f_0)	1.1303	1.0267	1.2339
Equilibrium Constant (M)	3.1294e-07	2.4106e-07	3.8482e-07

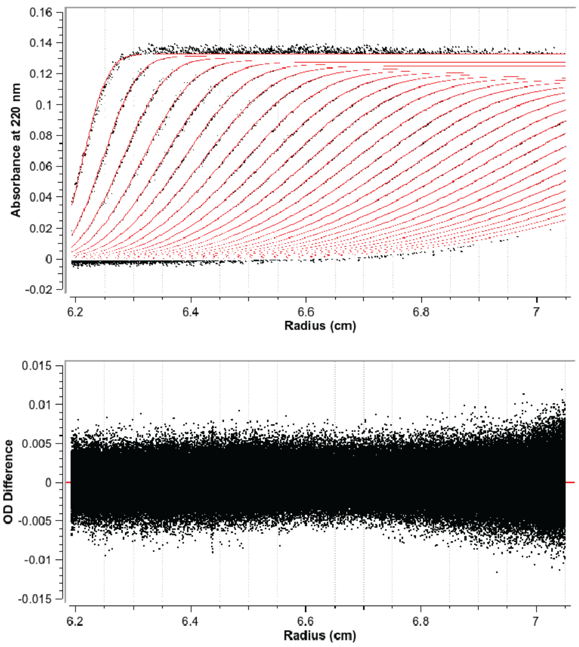

Supplementary Figure 1. Discrete Model Genetic Algorithm (DMGA) fit to SV-AUC data of 0.5 µM PARP15 ART. Top panel shows every tenth time- and radially-invariant noise corrected scan (black) and the model fit of the DMGA (red). Bottom panel shows the residuals of the fit.

Supplementary Table 3. Select details of an interface analysis of a PARP15 ART domain crystal dimer using the PISA server.[1]

PDB entry 6RY4	Chain A	Chain B
**No. Interface residues**	27 (13.7%)	27 (13.7%)
**Solvent accessible area (Å^2)**	1051.3 (9.9%)	1061.4 (9.9%)
**Gain of solvation energy on complex formation (kcal/mol)**	-4.8	-5.8

Interface details

#	Protomer 1	Dist. [Å]	Protomer 2
	**Hydrogen Bonds**		
**1**	B: GLN 543 [NE2]	3.11	A: PHE 532 [O]
**2**	B: GLN 543 [NE2]	3.06	A: GLN 535 [OE1]
**3**	B: GLN 543 [NE2]	3.60	A: SER 536 [OG]
**4**	B: ARG 576 [NH2]	2.94	A: ASN 575 [OD1]
**5**	B: ARG 576 [NE]	2.94	A: SER 577 [OG]
**6**	B: HIS 572 [NE2]	2.73	A: THR 644 [O]
**7**	B: ARG 576 [NH2]	2.97	A: ASP 665 [OD1]
**8**	B: ASP 665 [N]	2.76	A: ASP 665 [OD2]
**9**	B: ARG 576 [NH1]	2.92	A: ASP 665 [OD2]
**10**	B: PHE 532 [O]	3.04	A: GLN 543 [NE2]
**11**	B: GLN 535 [OE1]	2.96	A: GLN 543 [NE2]
**12**	B: SER 536 [OG]	3.66	A: GLN 543 [NE2]
**13**	B: ASN 575 [OD1]	2.86	A: ARG 576 [NH2]
**14**	B: SER 577 [OG]	2.73	A: ARG 576 [NE]
**15**	B: THR 644 [O]	2.58	A: HIS 572 [NE2]
**16**	B: ASP 665 [OD1]	2.96	A: ARG 576 [NH2]
**17**	B: ASP 665 [OD2]	2.74	A: ASP 665 [N]
**18**	B: ASP 665 [OD2]	3.11	A: ARG 576 [NH1]
	**Salt Bridges**		
**1**	B: ARG 576 [NH1]	3.75	A: ASP 665 [OD1]
**2**	B: ARG 576 [NH2]	2.97	A: ASP 665 [OD1]
**3**	B: ARG 576 [NH1]	2.92	A: ASP 665 [OD2]
**4**	B: ARG 576 [NH2]	3.56	A: ASP 665 [OD2]
**5**	B: ASP 665 [OD1]	3.74	A: ARG 576 [NH1]
**6**	B: ASP 665 [OD1]	2.96	A: ARG 576 [NH2]
**7**	B: ASP 665 [OD2]	3.11	A: ARG 576 [NH1]
**8**	B: ASP 665 [OD2]	3.67	A: ARG 576 [NH2]

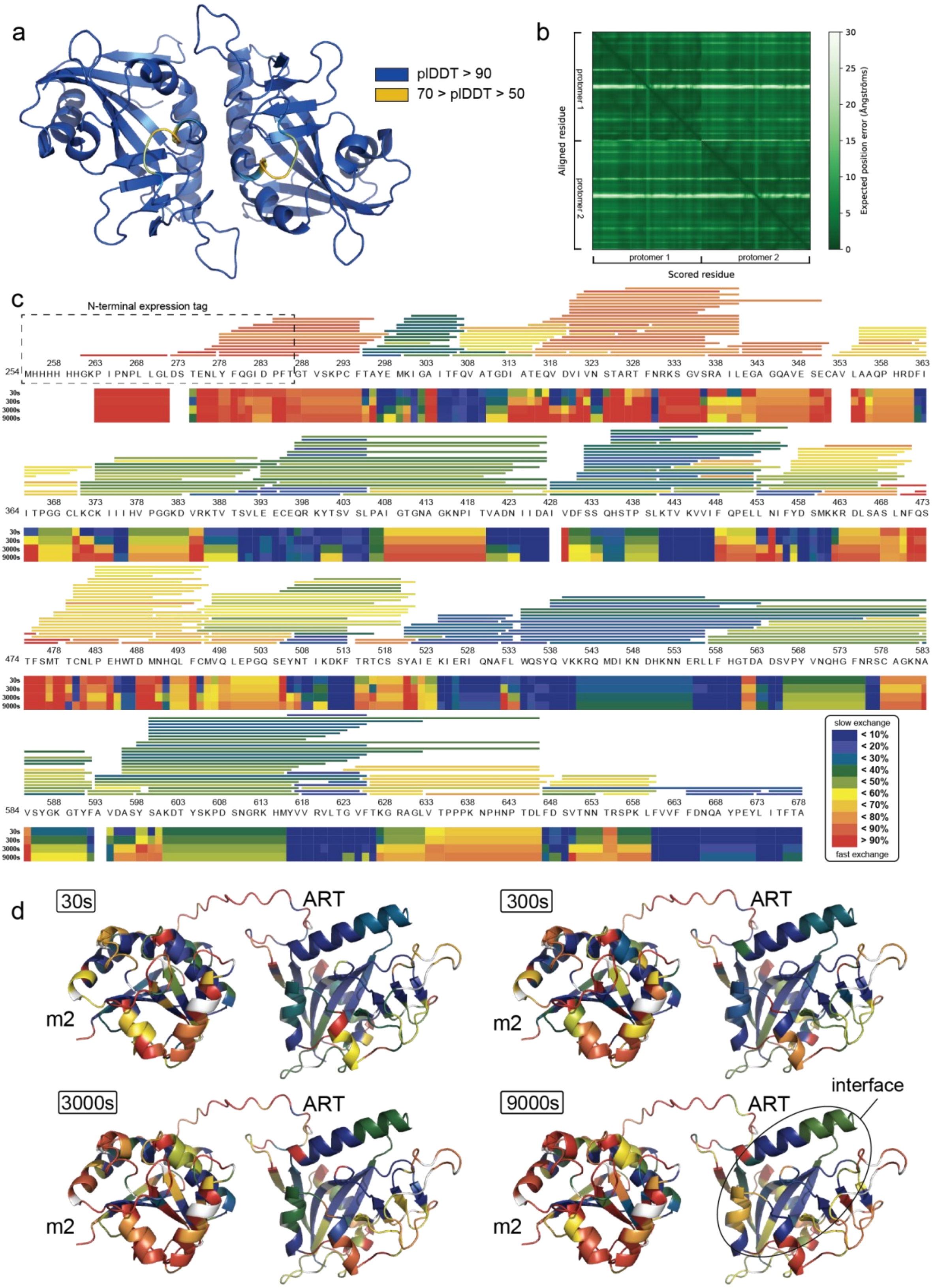

Legend – see following page.

Supplementary Figure 2. Details on the Alphafold3 model of the PARP15 ART domain dimer and HDX-MS data of the m2-ART dimer. Related to Figure 2. a pIDDT score mapped onto the highest-ranking model.[2] The two low-scoring regions represent the D-loop which is often not resolved in published crystal structures. **b** Predicted Aligned Error (PAE) plot.[2] **c** HDX-MS heatmap for PARP15 m2-ART, color code is based on a least-squares calculation of observed deuterium uptake without smoothing (see color key at the right). Cold color (slower exchange) indicates protection or secondary structure, whereas warm color represents fast exchange indicative of dynamic solvent accessible regions. Bars above the heatmap show the sequence coverage and are color coded to uptake averaged over the four time points. **d** Data from panel C superimposed on the Alphafold3 model of the m2-ART construct.[2] The dimer interface is indicated in the model at the bottom right. Source data are provided as a Source Data file.

Supplementary Table 4. BS3 crosslinked peptides and their detected masses. Please refer to Figure 2 and Supplementary Figure 3 for further explanation.

Seq Pos 1	PepSeq1	Link Pos 1	Seq Pos 2	PepSeq2	Link Pos 2	Score	z	Exp m/z	Calc m/z	Mass Error (ppm)
627	VLTGVFTKGR	8	586	NAVSYGK	5	11,9	3	651,696	651,698	-3,18
627	VLTGVFTKGR	8	586	NAVSYGK	5	10,64	2	977,040	977,044	-4,38
627	VLTGVFTKGR	8	586	NAVSYGK	5	8,8	3	651,695	651,698	-4,67
586	NAVSYGK	5	627	VLTGVFTKGR	8	10,56	3	651,696	651,698	-4,19
586	NAVSYGK	5	627	VLTGVFTKGR	8	9,51	3	651,699	651,698	1,66
586	NAVSYGK	5	627	VLTGVFTKGR	8	7,73	3	651,697	651,698	-2,01
627	VLTGVFTKGR	8	581	SC$_{CAM}$lAGKNAVSYGK	5	10,33	4	614,829	614,830	-0,92
627	VLTGVFTKGR	8	581	SC$_{CAM}$AGKNAVSYGK	5	9,32	4	614,826	614,830	-5,19
581	SC$_{CAM}$AGKNAVSYGK	5	627	VLTGVFTKGR	8	10,43	3	819,437	819,437	0,44
588	NAVSYGKGTYFAVD ASYSAK	7	581	SC$_{CAM}$AGKNAVSYGK	5	7,44	4	870,172	870,171	0,42
588	NAVSYGKGTYFAVD ASYSAK	7	581	SC$_{CAM}$AGKNAVSYGK	5	9,36	4	870,172	870,171	1,4
581	SC$_{CAM}$AGKNAVSYGK	5	588	NAVSYGKGTYFAVDASYSAK	7	8,72	4	870,168	870,171	-4,04
627	VLTGVFTKGR	8	551	NDHKNNER	4	8,48	4	561,047	561,049	-2,37
551	NDHKNNER	4	627	VLTGVFTKGR	8	7,72	4	561,046	561,049	-4,33

SeqPos: linked residue

PepSeq: peptide sequence

LinkPos: link position within peptide

z: charge

Exp m/z: experimental m/z ratio

Calc m/z: calculated m/z ratio

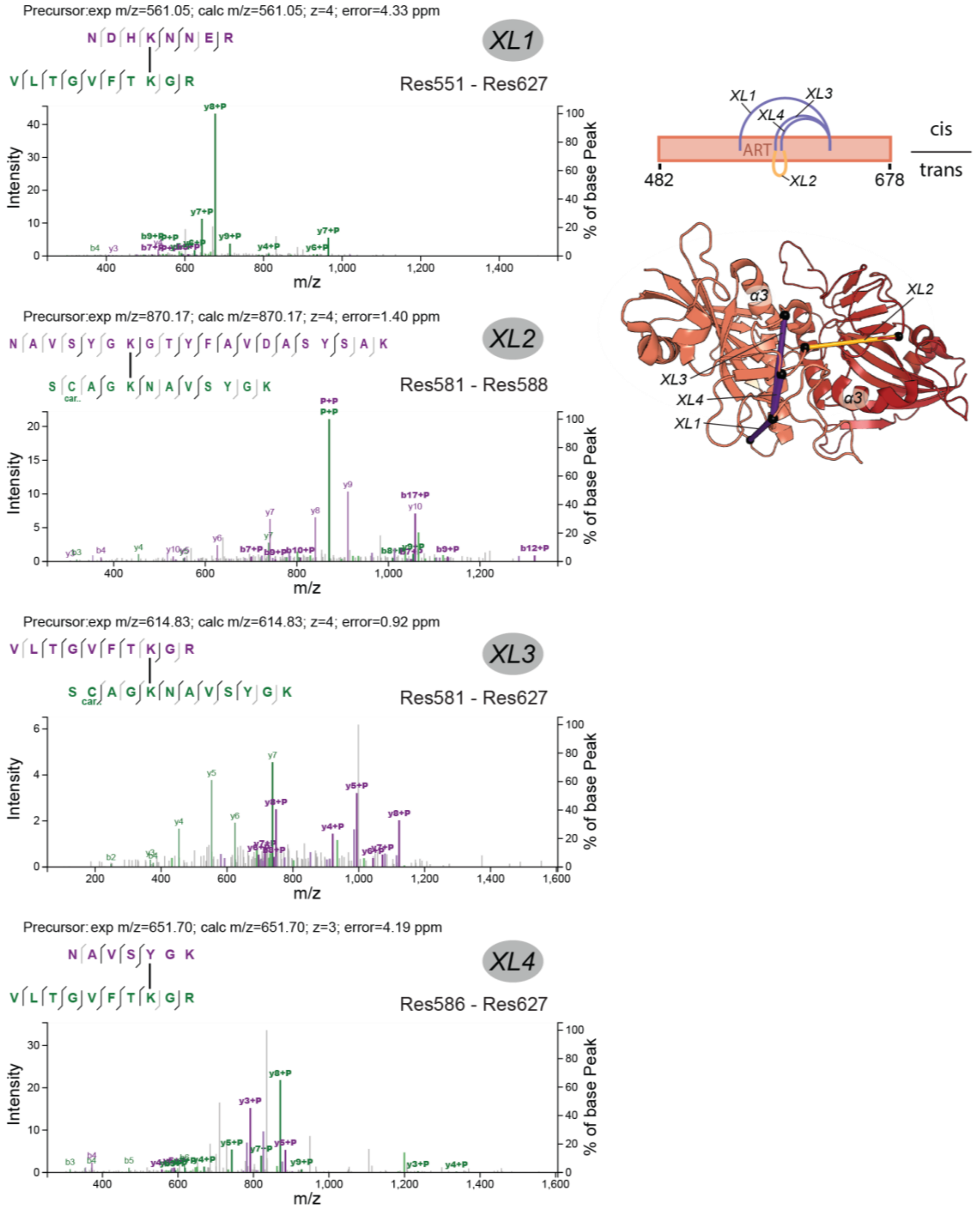

Supplementary Figure 3. Mass spectra of high-ranking cross-links from BS3-crosslinking of the PARP15 ART domain, related to Figure 2. Mass spectrometry fragmentation spectra of the four putative crosslinked peptides detected with high confidence. Intensities were plotted against the mass-to-charge (m/z) ratios, and the highlighted signals in the spectrum correlate to the detected Y- and B-ions. The sequence of each peptide is given above its mass spectrum, with the crosslinked residues connected by a line. Residue numbering according to full-length PARP15 (isoform 1). The schematic on the right illustrates the location of each crosslink on the PARP15 ART domain.

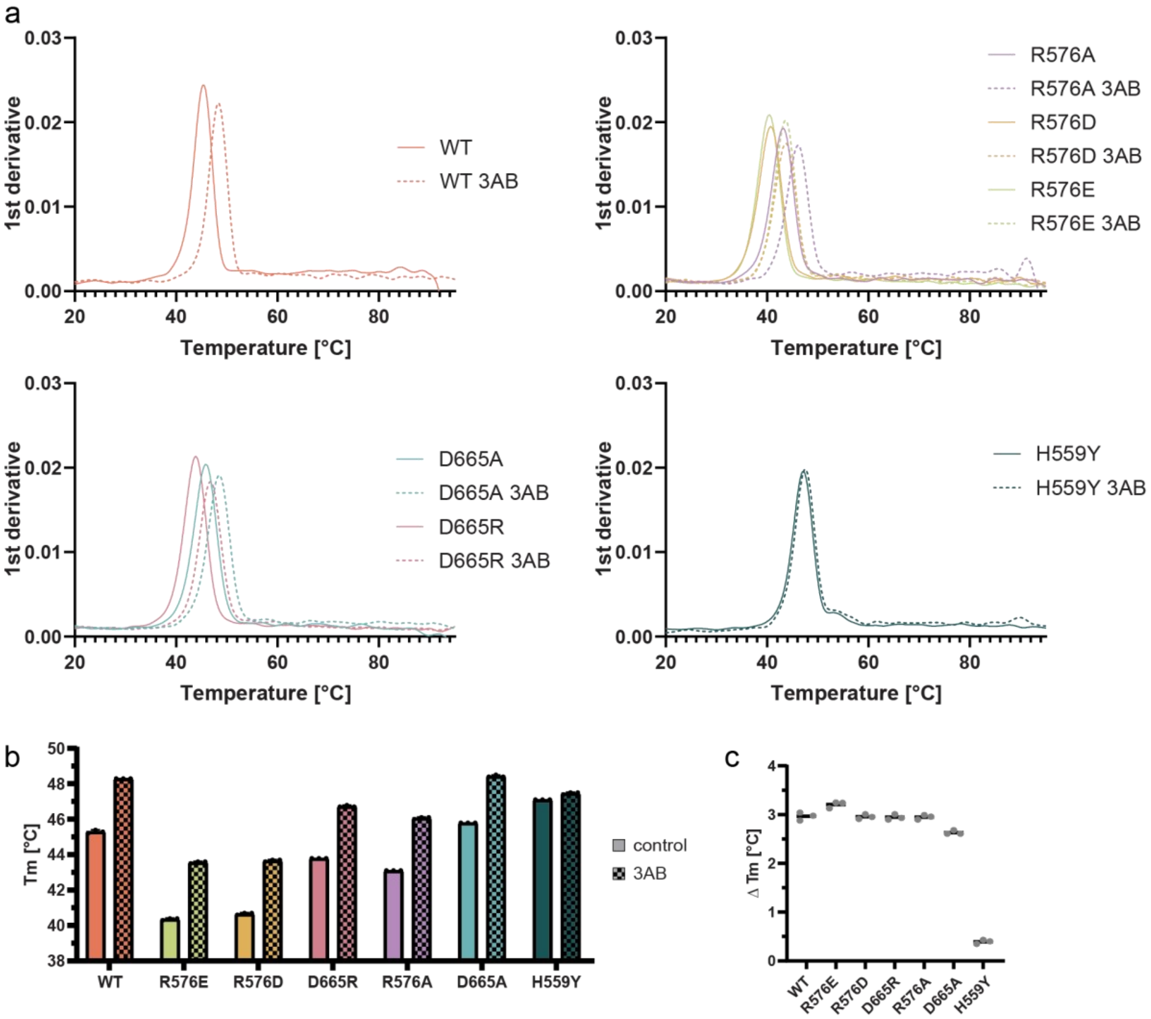

d

variant	Tm (apo)		Tm (3AB)	
	mean	SD	mean	SD
WT	45.3°C	0.09°C	48.3°C	0.03°C
R576E	40.4°C	0.04°C	43.6°C	0.03°C
R576D	40.7°C	0.06°C	43.6°C	0.05°C
D665R	43.8°C	0.02°C	46.8°C	0.08°C
R576A	43.1°C	0.02°C	46.0°C	0.06°C
D665A	45.8°C	0.03°C	48.4°C	0.02°C
H559Y	47.1°C	0.03°C	47.5°C	0.04°C

Supplementary Figure 4. Thermal stability analysis of ART domain dimer interface mutants by nanoDSF. a Averaged (n=3) first derivatives of transitions for each PARP15 ART domain variant, recorded at 0.2 mg/ml protein concentration. Variants include the wild-type ART domain, the dimer interface mutants, and the catalytically inactive H559Y mutant, each with and without the addition of 3-aminobenzamide (3AB). Most interface mutants were less stable than the wild-type ART domain but melted in symmetrical peaks. **b** Representation of the melting temperature (T_m) derived from panel (a). PARP15 dimerization contributed to thermal stability of the domain; however, monomeric mutants were stabilized by 3AB. **c** Shifts in the melting temperatures (ΔT_m) of wild type and mutant ART domain induced by 3AB. All interface mutants were stabilized to a similar degree; only the ligand-binding incompetent H559Y mutant was not notably stabilized by excess 3AB. Data are represented as n=3 replicates and mean. Derived from panel (b). **d** Summary of the mean melting temperatures and standard deviations (in degrees Celsius) derived from panel (b). Source data are provided as a Source Data file.

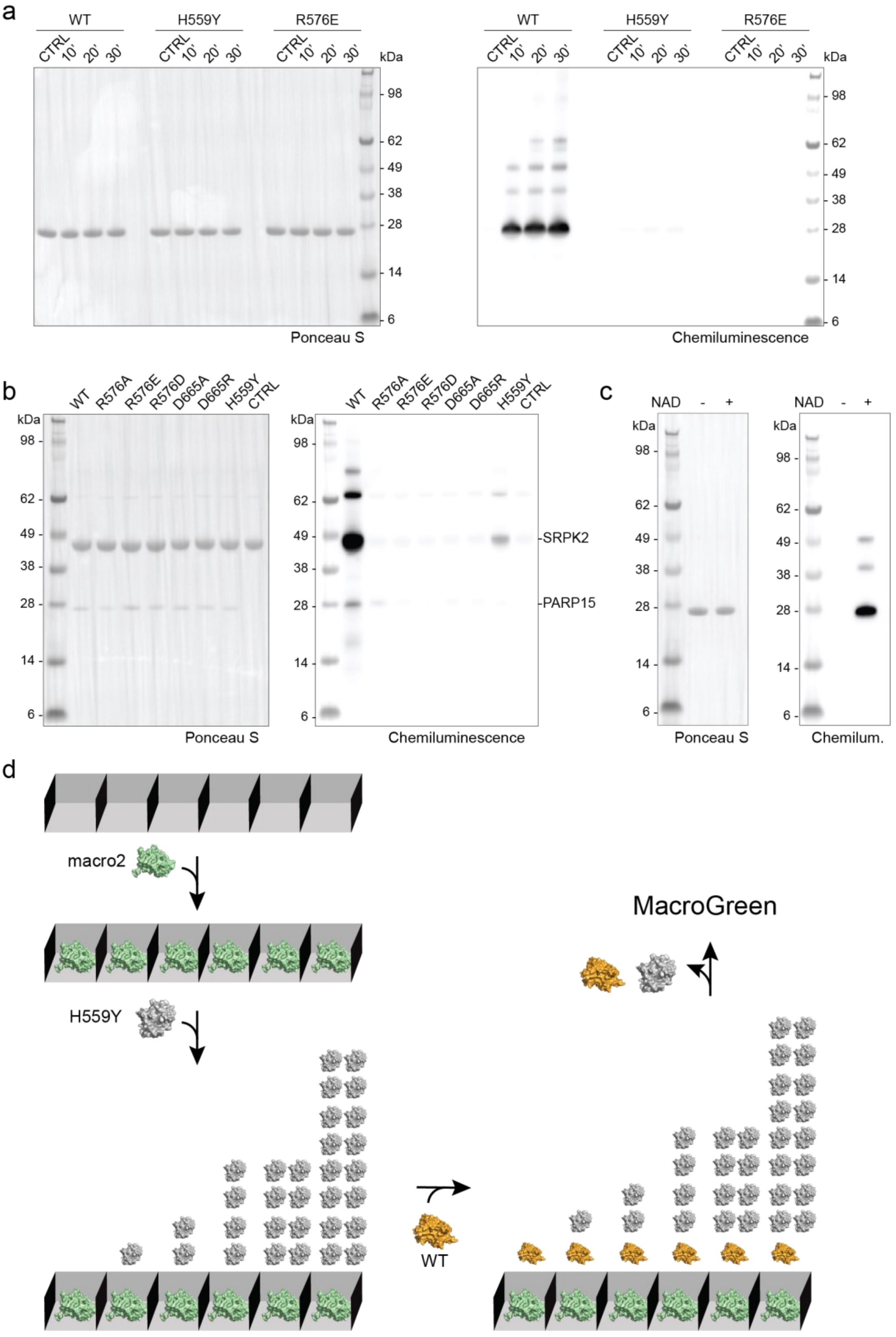

Legend – see following page.

Supplementary Figure 5. PARP15 *in vitro* activity assays. a Levels of pre-automodification of PARP15 ART domain originating during expression in *E. coli* were tested using a mono-ADP-ribose specific antibody (Bio-Rad) for the wild-type, the catalytically inactive H559Y mutant, and the dimer interface mutant R576E enzyme. NAD^+ (50 μM) was added, and the reactions were allowed to proceed for 10, 20, and 30 minutes. NAD^+ was omitted in the control reaction (CTRL). Ponceau S staining served as a loading control. **b** Trans-modification activity of PARP15 ART variants was tested in a Western blot assay using SRSF protein kinase 2 (SRPK2) as target. 50 μM NAD^+, spiked with 10% Biotin-NAD^+, was added to 1 μM PARP15 and 5 μM SRPK2. Biotin-ADP-ribose was detected with HRP-streptavidin. PARP15 was omitted in the control (CTRL). Ponceau S staining served as a loading control. **c** Western Blot confirms auto-MARylation on PARP15 ART after the SV-AUC run depicted in Figure 3g. Antibody: anti-MAR specific (Bio-Rad). Ponceau S staining served as a loading control. **d** Schematic of the assay performed in Figure 3f of the main text. High-binding plates coated with PARP15 macrodomain 2 were incubated with the catalytically inactive H559Y mutant (at a range of concentrations) and wild-type PARP15 (at a constant concentration of 250 nM). The ADP-ribosylation reaction was started by addition of NAD^+. After 40 minutes, NAD^+ and PARP15 WT and H559Y were washed off, and ADP-ribose on macrodomain 2 was detected using MacroGreen reagent.[3]

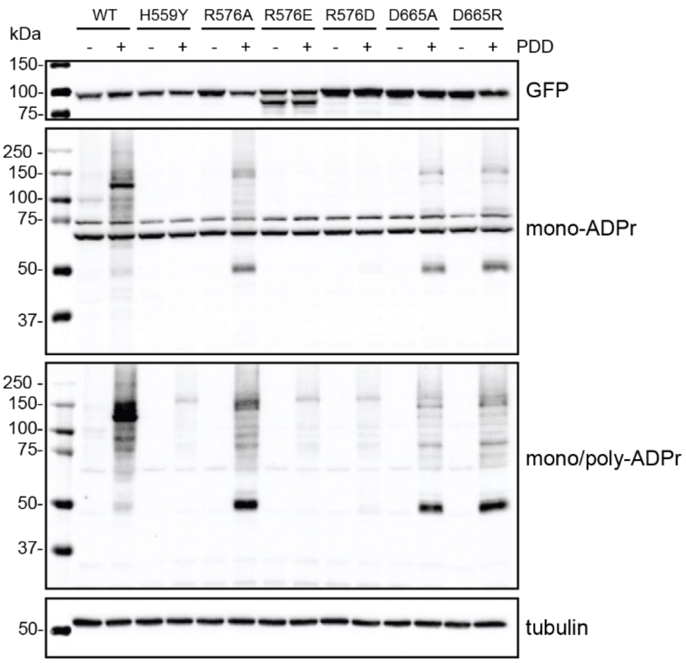

Supplementary Figure 6. The activity of PARP15 mutants expressed in HEK293T cells is reduced compared to wild-type PARP15, related to Figure 4. Each expression construct was tested in absence and presence of PARG inhibitor (PDD 00017273). PARP15 expression levels are probed with an anti-GFP antibody, tubulin staining serves as loading control. ADP-ribosylation levels were tested with mono-ADP-ribose specific and mono/poly-ADP-ribose specific antibodies. For details, please refer to the methods section Cellular ADP-ribosylation assay (Western blot).

Supplementary Table 5. Crystal parameters, data collection, and refinement statistics.
Related to Figure 5 and methods section X-ray Crystallography.

**Data collection**	**PARP15 ART DOMAIN MUTANT R576E (PDB: 9IFV)**		
Xray source	MAX IV beamline BioMAX		
Wavelength (Å)	0.97625		
Space group	C 2 2 2_1		
Unit cell dimensions			
a, b, c (Å)	75.71, 76.70, 155.56		
α, β, γ (°)	90, 90, 90		
	**Overall**	**Inner**	**Outer shell**
Low resolution limit (Å)	77.781	77.781	1.516
High resolution limit (Å)	1.431	4.237	1.431
Unique reflections	68482	3424	3424
R_{merge} (all I+ & I-)	0.112	0.116	1.573
R_{pim} (all I+ & I-)	0.032	0.034	0.592
Completeness, spherical (%)	82.0	99.9	26.2
Completeness, ellipsoidal (%)	91.4	99.9	57.0
Redundancy	12.3	12.7	7.7
Mean <I>/<σI>	11.2	19.9	1.4
CC(1/2)	0.994	0.987	0.796
**Refinement**			
Resolution (Å)	37.86 - 1.43		
R_{all} (%)	0.195		
R_{free} (%)	0.224		
No atoms			
Protein	6379		
Ligand/ion	36		
Water	217		
B-factors			
Protein	34.54		
Ligand/ion	25.89		
Water	35.94		
r.m.s.d. bond length (Å)	0.0148		
r.m.s.d. bond angle (°)	1.1697		
**Ramachandran plot**			
Most favoured (%)	98		
Allowed (%)	2		

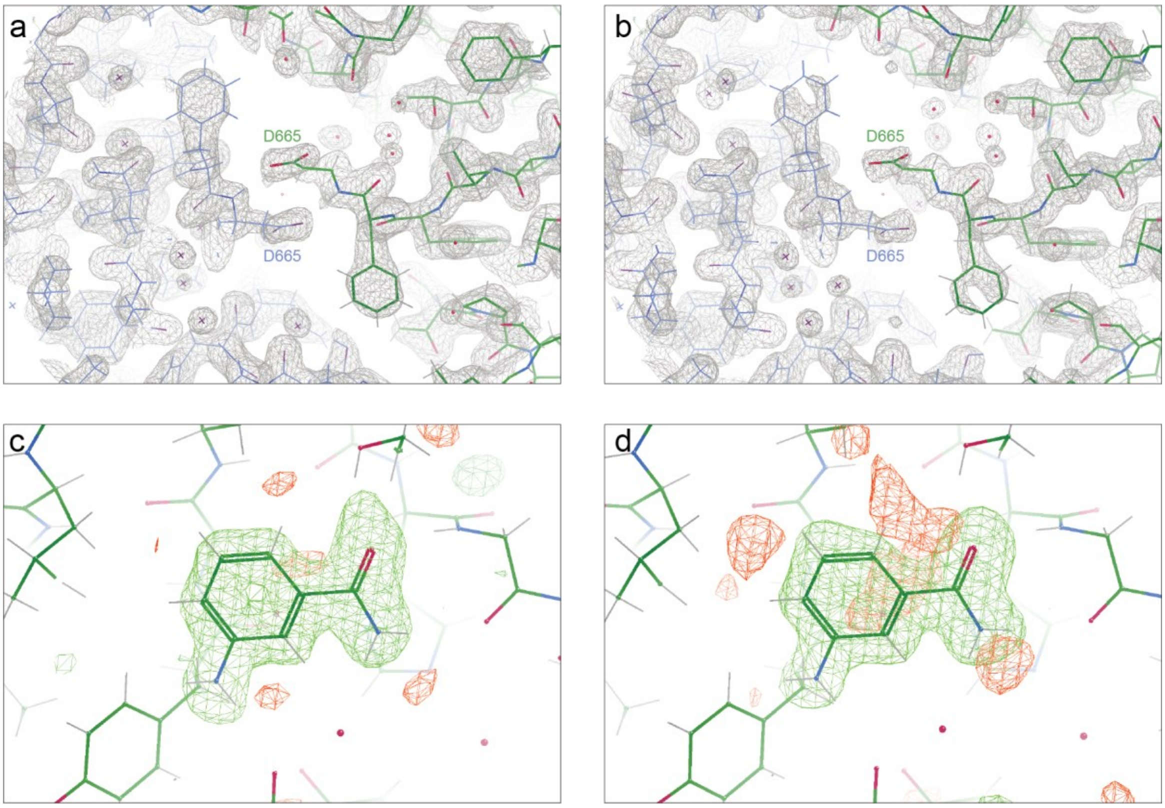

Supplementary Figure 7. Electron density validation of PARP15 ART domain mutant R576E crystal structure (PDB: 9IFV). a,b 2m|F_o|-D|F_c| electron density maps at the dimer interface formed by chain A (**a**) or chain B (**b**), contoured at 1.2σ. Asymmetric unit atoms are colored in green, with water molecules displayed as spheres, whereas symmetry related atoms are colored in blue, with water molecules displayed as crosses. **c,d** Omit maps (m|F_o|-D|F_c|, contoured at 3σ), calculated using the polder map function in Phenix[4] for ligand 3AB in chain A (**c**) or chain B (**d**). Positive omit difference density is displayed in green; negative omit difference density is displayed in red.

Supplementary Table 6. Interface analysis of the PARP15 ART domain mutant R576E crystal (PDB: 9IFV) using the PISA server. [1]

Chain A – Chain A	Chain A_1	Chain A_2
**No. Interface residues**	27 (13.6%)	27 (13.6%)
**Buried area (Å2)**	974.3 (9.2%)	977.8 (9.3%)
**Gain of solvation energy on complex formation (kcal/mol)**	-4.8	-4.8
**Chain B – Chain B**	**Chain B_1**	**Chain B_2**
**No. Interface residues**	26 (13.1%)	26 (13.1%)
**Solvent accessible area (Å2)**	953.5 (9.2%)	953.5 (9.2%)
**Gain of solvation energy on complex formation (kcal/mol)**	-6.0	-6.0

Interface details: **Chain A – Chain A**

#	Protomer 1	Dist. [Å]	Protomer 2
	**Hydrogen Bonds**		
**1**	A: GLN 543 [NE2]	3.09	A: PHE 532 [O]
**2**	A: GLN 543 [NE2]	2.84	A: GLN 535 [OE1]
**3**	A: GLN 543 [NE2]	3.40	A: SER 536 [OG]
**4**	A: HIS 572 [NE2]	2.71	A: THR 644 [O]
**5**	A: ASP 665 [N]	2.73	A: ASP 665 [OD2]
**6**	A: PHE 532 [O]	3.09	A: GLN 543 [NE2]
**7**	A: GLN 535 [OE1]	2.84	A: GLN 543 [NE2]
**8**	A: SER 536 [OG]	3.40	A: GLN 543 [NE2]
**9**	A: THR 644 [O]	2.71	A: HIS 572 [NE2]
**10**	A: ASP 665 [OD2]	2.73	A: ASP 665 [N]

Interface details: **Chain B – Chain B**

#	Protomer 1	Dist. [Å]	Protomer 2
	**Hydrogen Bonds**		
**1**	B: GLN 543 [NE2]	3.09	B: PHE 532 [O]
**2**	B: GLN 543 [NE2]	2.90	B: GLN 535 [OE1]
**3**	B: GLN 543 [NE2]	3.39	B: SER 536 [OG]
**4**	B: HIS 572 [NE2]	2.61	B: THR 644 [O]
**5**	B: ASP 665 [N]	2.65	B: ASP 665 [OD2]
**6**	B: PHE 532 [O]	3.09	B: GLN 543 [NE2]
**7**	B: GLN 535 [OE1]	2.90	B: GLN 543 [NE2]
**8**	B: SER 536 [OG]	3.39	B: GLN 543 [NE2]
**9**	B: THR 644 [O]	2.61	B: HIS 572 [NE2]
**10**	B: ASP 665 [OD2]	2.65	B: ASP 665 [N]

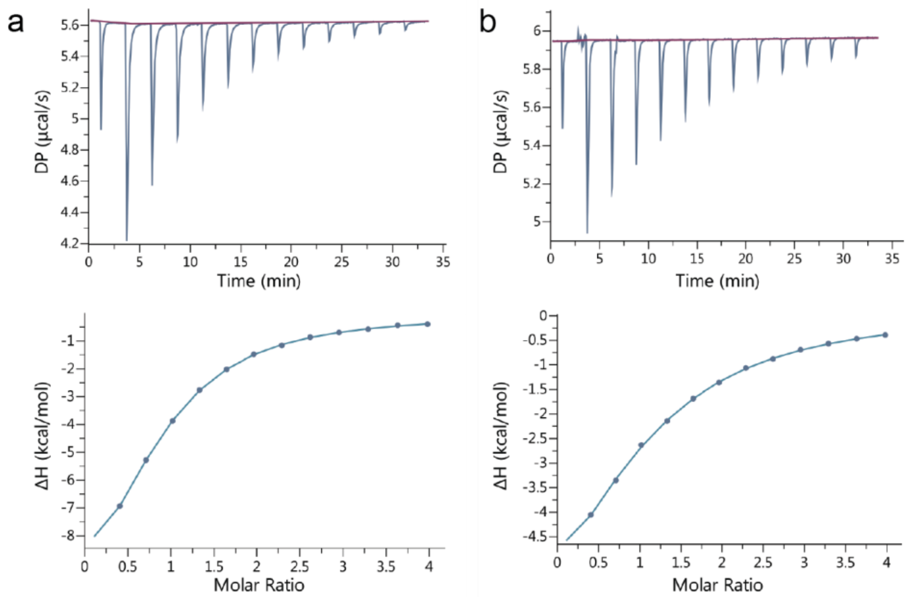

Supplementary Figure 8. EB-47 binding to WT PARP15 and R576E mutant, measured by isothermal titration calorimetry (ITC). ITC raw data (upper panel) and peak integrals as a function of molar ratio (lower panel) for 44 µM wild-type PARP15 (**a**) or 44 µM PARP15 R576E (**b**) at an EB-47 concentration of 1mM. PARP15 R576E has a reduced affinity for the ligand, but neither WT nor mutant PARP15 show evidence of binding cooperativity: K_d^{WT} = 23.9±1.0 µM; N = 0.98±0.01. K_d^{R576E} = 46.6±5.5 µM; N = 1.12±0.05.

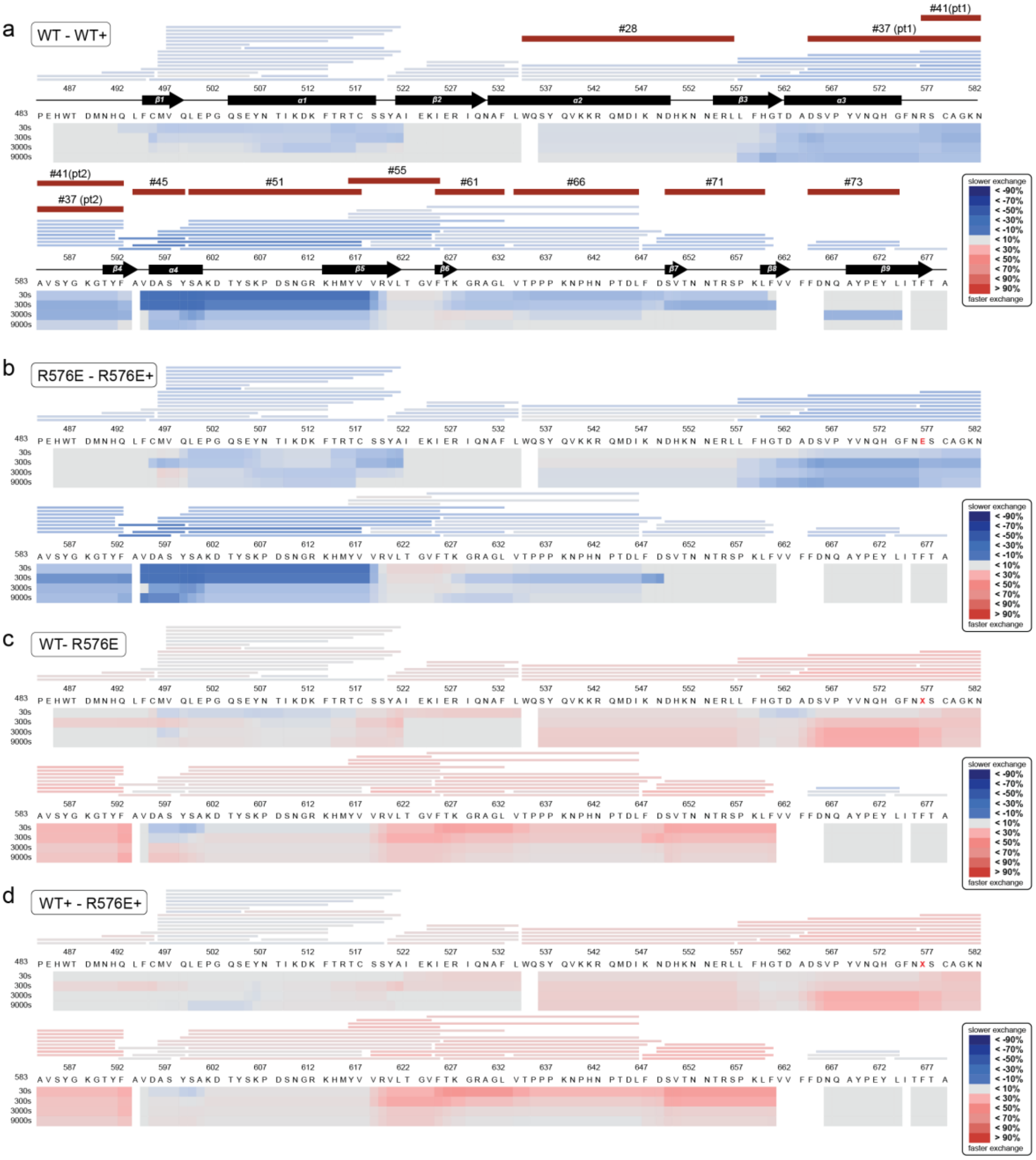

Supplementary Figure 9. HDX-MS difference plots illustrating the relative (de)protection of individual peptides, related to Figure 6. a Wild-type PARP15 in absence vs. in presence of EB-47. Secondary structure elements derived from crystal structures are depicted in black. Selected peptides shown in Figure 6 of the main text are depicted as dark red bars. **b** PARP15 R576E in absence vs. in presence of EB-47. **c** Wild-type PARP15 vs. PARP15 R576E, in absence of EB-47. **d** Wild-type PARP15 vs. PARP15 R576E, in presence of EB-47. Generally, blue colored peptides represent protection (slower deuterium exchange) whereas red colored peptides indicate de-protection (faster deuterium exchange) compared to the reference state. Source data are provided as a Source Data file.

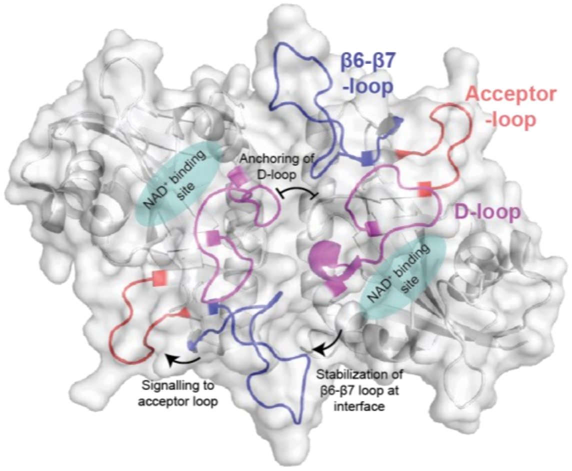

Supplementary Figure 10. Key loops affected by PARP15 dimerization, as observed in HDX-MS experiment shown in figure 6. The ART domain dimer is depicted in grey, with select loops in color. Dimerization of the domain leads to an anchoring of the D-loop base at the interface, which contributes to opening the NAD^+ binding site. The β6-β7 loop engages at the interface, relaying structural information to the acceptor loop positioned at the entry to the catalytic site.

Supplementary References

1. Krissinel E, Henrick K. Inference of macromolecular assemblies from crystalline state. *J Mol Biol* **372**, 774-797 (2007).

2. Abramson J*, et al.* Accurate structure prediction of biomolecular interactions with AlphaFold 3. *Nature* **630**, 493-500 (2024).

3. García-Saura, AG, Herzog LK, Dantuma NP, Schüler H. MacroGreen, a simple tool for detection of ADP-ribosylated proteins. *Commun Biol* **4**, 919 (2021).

4. Liebschner, D, Afonine, PV, Moriarty, NW, Poon, BK, Sobolev, OV, Terwilliger, TC & Adams, PD. Polder maps: improving OMIT maps by excluding bulk solvent. *Acta Cryst* **D73**, 148-157 (2017).
