# Supplementary material for: Regulation of ADP-ribosyltransferase activity by ART domain dimerization in PARP15
Source: Nat Commun. 2025 Oct 29;16:9567. doi: 10.1038/s41467-025-65315-9 (PMC12572374; doi:10.1038/s41467-025-65315-9)
Supplement: Supplementary file 2 — Reporting Summary [file 41467_2025_65315_MOESM2_ESM.pdf]

Corresponding author(s): Herwig Schüler
Last updated by author(s): Aug 13, 2025

Reporting Summary

Nature Portfolio wishes to improve the reproducibility of the work that we publish. This form provides structure for consistency and transparency in reporting. For further information on Nature Portfolio policies, see our Editorial Policies and the Editorial Policy Checklist.

Statistics

For all statistical analyses, confirm that the following items are present in the figure legend, table legend, main text, or Methods section.

n/a	Confirmed	
☐	☒	The exact sample size (*n*) for each experimental group/condition, given as a discrete number and unit of measurement
☐	☒	A statement on whether measurements were taken from distinct samples or whether the same sample was measured repeatedly
☐	☒	The statistical test(s) used AND whether they are one- or two-sided<br>*Only common tests should be described solely by name; describe more complex techniques in the Methods section.*
☒	☐	A description of all covariates tested
☐	☒	A description of any assumptions or corrections, such as tests of normality and adjustment for multiple comparisons
☐	☒	A full description of the statistical parameters including central tendency (e.g. means) or other basic estimates (e.g. regression coefficient) AND variation (e.g. standard deviation) or associated estimates of uncertainty (e.g. confidence intervals)
☐	☒	For null hypothesis testing, the test statistic (e.g. *F*, *t*, *r*) with confidence intervals, effect sizes, degrees of freedom and *P* value noted<br>*Give P values as exact values whenever suitable.*
☒	☐	For Bayesian analysis, information on the choice of priors and Markov chain Monte Carlo settings
☒	☐	For hierarchical and complex designs, identification of the appropriate level for tests and full reporting of outcomes
☒	☐	Estimates of effect sizes (e.g. Cohen's *d*, Pearson's *r*), indicating how they were calculated

Our web collection on statistics for biologists contains articles on many of the points above.

Software and code

Policy information about availability of computer code

Data collection	See methods section.
Data analysis	Softwares are referenced in the method section.<br><br>OMNISEC, Malvern Panalytical, Version 11.36<br>DiscoverMP, Refeyn, v2024 R2<br>Mascot Distiller, Version 2.8<br>Mascot Deamon, Version 2.8<br>xiSEARCH, Version 1.8.2<br>xiVIEW<br>PDBePISA EMBL-EBI, https://www.ebi.ac.uk/pdbe/pisa/<br>AlphaFold3, https://alphafoldserver.com/<br>PyMOL, Schrodinger, Version 2.5.8<br>ImageJ (FIJI), Version 1.54f<br>GraphPad Prism, GraphPad Software, Version 10.4.1<br>PR.ThermControl, NanoTemper Technologies, Version 2.0.4<br>Phenix, Version 1.21rc1_5084<br>autoPROC, Version 1.0.5<br>XDS, VERSION Jun 30, 2023 BUILT=20230630<br>AIMLESS, Version 0.7.9

STARANISO
Coot, Version 0.9.8.93
MicroCal PEAQ-ITC Analysis Software, Malvern Panalytical, Version 1.41
HDExaminer, LEAP Ltd., Version 3.4.2
UltraScan III (ver. 4)

For manuscripts utilizing custom algorithms or software that are central to the research but not yet described in published literature, software must be made available to editors and reviewers. We strongly encourage code deposition in a community repository (e.g. GitHub). See the Nature Portfolio guidelines for submitting code & software for further information.

Data

Policy information about availability of data

All manuscripts must include a data availability statement. This statement should provide the following information, where applicable:

- Accession codes, unique identifiers, or web links for publicly available datasets
- A description of any restrictions on data availability
- For clinical datasets or third party data, please ensure that the statement adheres to our policy

HDX-MS and crosslinking-MS data have been deposited to the PRIDE database (accession codes: PXD061403 and pending) and are publicly available as of the date of publication. The crystallographic structure of the PARP15 catalytic domain mutant (R576E) in complex with 3-aminobenzamide generated in this study has been deposited in the Protein Data Bank under accession code 9IFV. Source Data are provided with this paper. All additional data are available within the main text or the supplementary figures and tables.

Research involving human participants, their data, or biological material

Policy information about studies with human participants or human data. See also policy information about sex, gender (identity/presentation), and sexual orientation and race, ethnicity and racism.

Reporting on sex and gender	*Use the terms sex (biological attribute) and gender (shaped by social and cultural circumstances) carefully in order to avoid confusing both terms. Indicate if findings apply to only one sex or gender; describe whether sex and gender were considered in study design; whether sex and/or gender was determined based on self-reporting or assigned and methods used. Provide in the source data disaggregated sex and gender data, where this information has been collected, and if consent has been obtained for sharing of individual-level data; provide overall numbers in this Reporting Summary. Please state if this information has not been collected. Report sex- and gender-based analyses where performed, justify reasons for lack of sex- and gender-based analysis.*
Reporting on race, ethnicity, or other socially relevant groupings	*Please specify the socially constructed or socially relevant categorization variable(s) used in your manuscript and explain why they were used. Please note that such variables should not be used as proxies for other socially constructed/relevant variables (for example, race or ethnicity should not be used as a proxy for socioeconomic status). Provide clear definitions of the relevant terms used, how they were provided (by the participants/respondents, the researchers, or third parties), and the method(s) used to classify people into the different categories (e.g. self-report, census or administrative data, social media data, etc.) Please provide details about how you controlled for confounding variables in your analyses.*
Population characteristics	*Describe the covariate-relevant population characteristics of the human research participants (e.g. age, genotypic information, past and current diagnosis and treatment categories). If you filled out the behavioural & social sciences study design questions and have nothing to add here, write "See above."*
Recruitment	*Describe how participants were recruited. Outline any potential self-selection bias or other biases that may be present and how these are likely to impact results.*
Ethics oversight	*Identify the organization(s) that approved the study protocol.*

Note that full information on the approval of the study protocol must also be provided in the manuscript.

Field-specific reporting

Please select the one below that is the best fit for your research. If you are not sure, read the appropriate sections before making your selection.

☒ Life sciences ☐ Behavioural & social sciences ☐ Ecological, evolutionary & environmental sciences

For a reference copy of the document with all sections, see nature.com/documents/nr-reporting-summary-flat.pdf

Life sciences study design

All studies must disclose on these points even when the disclosure is negative.

Sample size	All biochemical assays were performed in triplicate or quadruplets. For analysis of immunofluorescence data, non-transfected cells were largely removed based on intensity thresholding (green channel - mEGFP); sample size represents all remaining cells (unequal for the different groups).

Data exclusions	For analysis of immunofluorescence data, non-transfected cells were largely removed based on intensity thresholding (green channel - mEGFP). Reason for that is that non-transfected cells in the sample have a big contribution to the data analysis, biasing it towards low expression and therefore enzyme activity. Especially for visualization the effects are detrimental. No outliers were removed in this study.
Replication	All experiments were performed at least twice in the same set-up, and more often in similar set-ups to ensure reproducibility. This is also true for mass photometry experiments, which were performed on an older, no longer available version of the equipment, thus limiting reproducibility. Immunofluorescence experiments were performed in biological triplicates, with slight modifications in the dosing conditions used. One representative condition was chosen for visualization and analysis.
Randomization	No randomization was performed in this study.
Blinding	No blinding was conducted during this study.

Reporting for specific materials, systems and methods

We require information from authors about some types of materials, experimental systems and methods used in many studies. Here, indicate whether each material, system or method listed is relevant to your study. If you are not sure if a list item applies to your research, read the appropriate section before selecting a response.

Materials & experimental systems

n/a	Involved in the study	
☐	☒	Antibodies
☐	☒	Eukaryotic cell lines
☒	☐	Palaeontology and archaeology
☒	☐	Animals and other organisms
☒	☐	Clinical data
☒	☐	Dual use research of concern
☒	☐	Plants

Methods

n/a	Involved in the study	
☒	☐	ChIP-seq
☒	☐	Flow cytometry
☒	☐	MRI-based neuroimaging

Antibodies

Antibodies used	Poly/Mono-ADP Ribose antibody (Rabbit monoclonal; 1:1000) (E6F6A), Cell Signaling Technology, 83732, RRID:AB_2749858;<br>Donkey anti-Rabbit IgG (H+L) Highly Cross-Adsorbed Secondary Antibody, Alexa Fluor™ 568 (1:1000), Invitrogen, A10042, RRID:AB_2534017;<br>α-Tubulin (Mouse monoclonal; 1:2000), Cell Signaling Technology, 3873, RRID:AB_1904178;<br>Anti-GFP, PABG1 (Rabbit polyclonal; 1:1000), Chromotek, PABG1-100; RRID:AB_2749857;<br>Mono-ADP-Ribose (2 μg/ml), clone AbD33204, Bio-Rad, HCA354;<br>Goat anti-rabbit IgG (1:10000), HRP linked, Invitrogen, 65-6120, RRID:AB_2533967;<br>Goat anti-mouse IgG (1:5000), HRP linked, Invitrogen; 62-6520, RRID:AB_2533947
Validation	All antibodies were validated by the manufacturers. We did not perform further validation.

Eukaryotic cell lines

Policy information about cell lines and Sex and Gender in Research

Cell line source(s)	HEK 293T (H. sapiens), ATCC, CRL-3216, RRID:CVCL_0063
Authentication	No further validation performed.
Mycoplasma contamination	All cell lines were tested for mycoplasma contamination on a regular basis.
Commonly misidentified lines<br>(See ICLAC register)	No commonly misidentified lines were used in this study.

Plants

Seed stocks	*Report on the source of all seed stocks or other plant material used. If applicable, state the seed stock centre and catalogue number. If plant specimens were collected from the field, describe the collection location, date and sampling procedures.*
Novel plant genotypes	*Describe the methods by which all novel plant genotypes were produced. This includes those generated by transgenic approaches, gene editing, chemical/radiation-based mutagenesis and hybridization. For transgenic lines, describe the transformation method, the number of independent lines analyzed and the generation upon which experiments were performed. For gene-edited lines, describe the editor used, the endogenous sequence targeted for editing, the targeting guide RNA sequence (if applicable) and how the editor was applied.*
Authentication	*Describe any authentication procedures for each seed stock used or novel genotype generated. Describe any experiments used to assess the effect of a mutation and, where applicable, how potential secondary effects (e.g. second site T-DNA insertions, mosiacism, off-target gene editing) were examined.*
